# Acceptance of Commercially Available Wearable Activity Trackers Among Adults Aged Over 50 and With Chronic Illness: A Mixed-Methods Evaluation

**DOI:** 10.2196/mhealth.4225

**Published:** 2016-01-27

**Authors:** Kathryn Mercer, Lora Giangregorio, Eric Schneider, Parmit Chilana, Melissa Li, Kelly Grindrod

**Affiliations:** ^1^ School of Pharmacy Faculty of Science University of Waterloo Waterloo, ON Canada; ^2^ Department of Kinesiology Faculty of Applied Health Sciences University of Waterloo Waterloo, ON Canada; ^3^ School of Pharmacy Faculty of Science Wingate University Wingate, NC United States; ^4^ Management Sciences Faculty of Engineering University of Waterloo Waterloo, ON Canada

**Keywords:** chronic disease, physical activity, sedentary lifestyle, wearables

## Abstract

**Background:**

Physical inactivity and sedentary behavior increase the risk of chronic illness and death. The newest generation of “wearable” activity trackers offers potential as a multifaceted intervention to help people become more active.

**Objective:**

To examine the usability and usefulness of wearable activity trackers for older adults living with chronic illness.

**Methods:**

We recruited a purposive sample of 32 participants over the age of 50, who had been previously diagnosed with a chronic illness, including vascular disease, diabetes, arthritis, and osteoporosis. Participants were between 52 and 84 years of age (mean 64); among the study participants, 23 (72%) were women and the mean body mass index was 31 kg/m^2^. Participants tested 5 trackers, including a simple pedometer (Sportline or Mio) followed by 4 wearable activity trackers (Fitbit Zip, Misfit Shine, Jawbone Up 24, and Withings Pulse) in random order. Selected devices represented the range of wearable products and features available on the Canadian market in 2014. Participants wore each device for at least 3 days and evaluated it using a questionnaire developed from the Technology Acceptance Model. We used focus groups to explore participant experiences and a thematic analysis approach to data collection and analysis.

**Results:**

Our study resulted in 4 themes: (1) adoption within a comfort zone; (2) self-awareness and goal setting; (3) purposes of data tracking; and (4) future of wearable activity trackers as health care devices. Prior to enrolling, few participants were aware of wearable activity trackers. Most also had been asked by a physician to exercise more and cited this as a motivation for testing the devices. None of the participants planned to purchase the simple pedometer after the study, citing poor accuracy and data loss, whereas 73% (N=32) planned to purchase a wearable activity tracker. Preferences varied but 50% felt they would buy a Fitbit and 42% felt they would buy a Misfit, Jawbone, or Withings. The simple pedometer had a mean acceptance score of 56/95 compared with 63 for the Withings, 65 for the Misfit and Jawbone, and 68 for the Fitbit. To improve usability, older users may benefit from devices that have better compatibility with personal computers or less-expensive Android mobile phones and tablets, and have comprehensive paper-based user manuals and apps that interpret user data.

**Conclusions:**

For older adults living with chronic illness, wearable activity trackers are perceived as useful and acceptable. New users may need support to both set up the device and learn how to interpret their data.

## Introduction

### Background

Physical activity levels often decline with age and over two thirds of adults over the age of 60 sit for more than 8.5 hours of their waking day [1]. Physical activity can improve blood pressure, body composition, and overall health, which can prevent frailty and can contribute to a longer independent life [2,3]. Physical activity guidelines recommend adults over the age of 50 to perform moderate to vigorous physical activity for at least 150 minutes/week [4-6]. The guidelines define inactivity as “insufficient amounts of moderate to vigorous physical activity” and Katzmarzyk has noted that this accounts for only 3% of waking hours [4-7]. However, a sedentary lifestyle and physical inactivity are independent risk factors for chronic disease and a shortened lifespan [8-10]. Sedentary behavior is “any waking behaviour characterized by an energy expenditure of 1.5 or less metabolic equivalents while in a sitting or reclining posture” and includes sitting, television watching, computer use, and travel [11].

Home-based physical activity programs have significant potential for encouraging physical activity in older adults [12]. Yet, although sedentary persons can certainly adopt and continue regular long-term physical activity [13], daily adherence to home-based programs is low [14]. Successful interventions are often expensive and time intensive [15]. Programs that build self-awareness may be most amenable to lasting participation [15]. Goal setting has significant promise when it comes to promoting physical and dietary changes [16]. There is evidence that other behavior change techniques, such as self-monitoring, risk communication, and the use of social support can be beneficial additions to change-based interventions [17]. There is also some evidence that tailored feedback and information helps adults adopt physical activity and health-based activities [18-20].

The main challenge to implementing many of the evidence-based physical activity interventions and behavior change techniques is that they are resource intensive. In most cases, often multiple individuals are required to create tailored programs for individual patients, provide education, and then follow-up to promote adherence [21]. Where resources are scarce and demand is high, it would be ideal to have a simple, inexpensive, patient-managed intervention that incorporates behavior change techniques and can help patients improve and maintain activity levels.

### Wearable Activity Trackers

Wearable activity trackers are a rapidly growing health-focused industry. There are many terms for the trackers, including *electronic activity monitors*, *fitness trackers*, *wearable activity monitors*, and *wearables*, but generally speaking, wearable activity trackers are devices that use sensors to help users automatically track step counts while aiming for a particular step count or activity goal. Pedometers are simple tools to promote self-awareness and self-monitoring of activity levels. Pedometer-based walking programs improve physical activity, body mass index, and blood pressure, even in adults who are already moderately active [22-26]. Improvements are typically greatest in programs that incorporate goals such as 10,000 steps daily [19].

The newest generation of pedometer is known as the wearable activity tracker. Similar to the behavioral strategies used in walking programs, the new trackers promote goal setting, self-efficacy, and tailored feedback through companion mobile apps and websites. The trackers do this by providing visual representations of activity data (step counts, altimetry, calories, sleep) over days and weeks. Some wearable activity trackers can also be used with social networking sites and other lifestyle apps for diet and stress, such as MyFitnessPal, Weight Watchers, and SparkPeople.

Although the new trackers have been designed as wellness devices rather than health or medical devices, they have considerable potential for use in health care. The BodyMedia FIT and Fitbit Zip trackers have been shown to provide valid measures of daily energy expenditures [27-30]. However, activity trackers may be less accurate for older adults with a shuffling, abnormal, or slow gait caused by conditions such as stroke or Parkinson’s disease [31-34]. There is growing evidence that these trackers do improve step count, and while there is acknowledged potential, there is currently little evidence yet that they improve health outcomes such as blood pressure or blood sugar [35-38].

### Persuasive Activity Trackers

The following conditions are necessary for persuasive technology to promote a behavior: motivation, physical ability, and an effective trigger [36]. Persuasive fitness technologies are attractive because they “automate” behavior change [39]. They offer convenient data collection, analysis, and storage over long periods with immediate automated feedback. Game-based features also offer points, leader boards, badges, trophies, vibrations, and progress bars to promote self-competition and building support and competitive networks, with the end result of encouraging and increasing physical activity [40,41].

Research has been emerging on the design and evaluation of persuasive technologies to promote physical activity. A matched case-control study with individuals who were members of the 10,000 steps Australia program found that individuals invited to use a website or mobile phone app were four times more likely to log their steps and 20 times more likely to achieve 10,000 daily steps [42]. Mobile phone (and app) developers are also starting to build pedometer functions into devices and using the phones’ main screen wallpapers to promote specific behaviors. For example, UbiFit Garden uses images of flowers (activities) and butterflies (goals) on the background screen of a user’s mobile phone to promote physical activity [43].

Wearable trackers have been increasingly identified as a tool for helping consumers prevent disease and increase physical activity [44,45]. Wearable activity monitors contain a wide range of behavior change techniques that have been often used in clinical behavioral interventions. In 2014, Lyon et al [46] identified 13 fitness trackers that provided tools for self-monitoring, feedback, and environmental change as well as other common behavior change techniques such as goal setting, self-monitoring, and feedback content that closely matched recommendations from social cognitive theory.

Emerging research into persuasive technology and wearable trackers offers significant promise for improving health and fitness. Research into better understanding how their design affects activity and behavior, how visualization helps both motivate and provide awareness, and how feedback can be better understood and used is a growing field [47-50]. Michie et al [51] discussed the wide range of approaches that can result in behavior change [51]. In 2003, Fogg [52] discussed how persuasive technologies employ a wide variety of strategies that influence behavior and activities, most notably drawing out self-monitoring and conditioning [52]. Self-monitoring is one of the most prevalent persuasive technologies; however, the most successful technologies often employ multiple strategies [53].

To date we know little about how older adults perceive new and emerging mobile health (mHealth) tools that include wearable activity trackers. While some studies conclude that health interventions that use technology are less effective than in-person interventions, there is limited evidence to support this conclusion [54-56]. Therefore, there is great potential in building a better understanding of mobile tools that can help older adults become more active on their own time, in their own space. Our objective was to examine the usability and usefulness of wearable activity trackers for older adults living with chronic illness as a first step to better understand how wearable fitness trackers can help older adults become healthier.

## Methods

### Design and Setting

Our research design was inspired by a similar study on mobile medication management apps [57]. We used a mixed-methods approach to examine the perceived acceptability of popular wearable technologies designed to promote fitness and healthy living. The research was conducted at the University of Waterloo School of Pharmacy. We qualitatively assessed user acceptance using a thematic analysis, which is reported according to the consolidated criteria for reporting qualitative research [58]. We also developed a technology acceptance questionnaire based on the Technology Acceptance Model (TAM) [59]. We did not develop any of the devices we tested and received ethical approval from the University of Waterloo Office of Research Ethics (Certificate Number 19440).

We made the following assumptions: (1) most older adults are not using wearable activity trackers; (2) many have chronic illnesses that would benefit from increased physical activity; (3) wearable technologies have the potential to improve activity levels; and (4) there are age-related barriers unique to older users. We also assumed that most older adults are not early adopters and we were therefore asking participants to assume the early adopter role for the duration of this study.

### Wearable Activity Trackers

To assess acceptability of commercially available wearable activity trackers, we began by identifying all devices available to Canadian consumers as of November 1, 2013. We identified and reviewed 4 devices by Fitbit, 2 devices by Jawbone, and 1 device each from Withings, Misfit, and Nike. The research team selected 4 devices for testing purpose, with each device representing a different feature available with activity trackers (Table 1). All 4 devices used an accelerometer to assess steps (pedometer). We chose the Fitbit Zip because it was inexpensive, could be clipped to clothing, and allowed users to track activity on a simple interactive screen. We selected the Jawbone Up 24 as it could be worn on the wrist and could collect data on sleep quality. We selected the Misfit Shine because it could be worn on the wrist or clothing, was waterproof, and could double as a watch. Finally, we selected the Withings Pulse because it could capture heart rate data.

### User Testing

#### Participants and Sampling Frame

We recruited individuals from local public libraries, community centers, and primary care clinics. We also posted information on community message boards and approached the organizers of public programs (eg, Active Seniors at the Kitchener Public Library) to allow us to make brief presentations at events targeting individuals over the age of 50. The participant characteristics are presented in Table 2.

**Table 1:** Comparison of the available features of wearable activity tracker devices assessed between January 1, 2014, and May 31, 2014.

	Fitbit Zip	Jawbone Up 24	Misfit Shine	Withings Pulse
Pedometer (steps)	X	X	X	X
Altimeter (stairs)				X
Waterproof			X	
Heart rate				X
Displays number of steps taken on device	X			X
Displays proportion of steps taken toward the total goal			X	
Default activity goal	10,000 steps	10,000 steps	1000 points (equivalent to 10,000 steps)	10,000 steps
Notifies the user of every 2000 steps		X		
Cost	US $60	US $150	US $130	US $100
Apple iOS	X	X	X	X
Android	X	X^a^	X	X

^a^Made available March 2014.

#### Procedure

Participants started by using a basic pedometer for 3 days. Participants were then provided 4 wearable activity trackers (Fitbit Zip, Jawbone Up 24, Misfit Shine, and Withings Pulse) in random order and were asked to use each device for at least 3 days. Participants received each new device from investigators and we assisted them in setting the device up if needed. Participants were not required to own their own mobile phone or tablet. A total of 12 participants did not own a mobile phone or tablet, and they were lent one from the investigators or shared one with a friend or family member. Participants were instructed to wear the activity tracker as intended by the manufacturer (eg, Fitbit Zip during waking hours, whereas Jawbone Up 24 during waking and sleeping hours). Participants were asked to synchronize the device and their tablet or mobile phone at least once during each trial period and were expected to record their data, specifically the number of steps captured each day. The purpose of collecting step count data was to ensure that participants could access the information rather than to assess the impact of the devices on their activity levels.

#### Data Collection and Analysis

Prior to testing any devices, a researcher measured each participant’s weight, height, resting heart rate, and blood pressure. Participants completed a paper-based questionnaire on demographics and computer experience. Participants self-reported their physical activity using the validated Short Form International Physical Activity Questionnaire, which assesses the duration and frequency of walking, moderate activity, and vigorous activity among adults [61]. High physical activity was defined as either a minimum of 1500 metabolic equivalent of task (MET) minutes/week of vigorous activity or a minimum of 3000 MET minutes/week. Moderate physical activity was defined as a minimum of 600 MET minutes/week, 3 or more days of vigorous activity of at least 20 minutes/day or 5 or more days of moderate activity or walking of at least 30 minutes/day. Individuals who did not meet the criteria for moderate or high activity were classified as having “low activity.”

After testing each device, participants completed a questionnaire to describe how they used the device, how satisfied they were with the device, whether or not they would purchase a device, and then rated the devices with a 17-item questionnaire developed using the TAM, which assesses the domains of external variables, perceived usefulness, perceived ease of use, attitude toward using, behavioral intention to use, and actual system use (Table 3) [62-65]. After testing all 5 devices, participants completed a final debrief questionnaire where they ranked the devices according to preference and indicated whether they planned to purchase a particular device. Demographic and clinical information was summarized using descriptive statistics using SPSS (version 22; IBM Corporation, New York, NY, USA).

**Table 2:** Participant characteristics^a^ (N=32).

Characteristic		Number (% or range)
Age		64 (52-84)
**Gender**		
	Male	9 (28)
	Female	23 (72)
**Medical condition(s)**		
	High cholesterol	10 (31)
	High blood pressure	17 (53)
	Stroke	1 (3)
	Prediabetes	4 (13)
	Type 2 diabetes	3 (9)
	Osteoarthritis	15 (47)
	Rheumatoid or inflammatory arthritis	3 (9)
	Chronic low back pain	1 (3)
Body mass index (kg/m^2^, mean)		31 (21-51)
**Blood pressure (mean)**		
	Systolic	129 (93-182)
	Diastolic	79 (67-95)
Heart rate (mean)		70 (50-98)
**Physical activity (mean)**		
	Low	2 (6)
	Moderate	21 (66)
	High	8 (25)
**Family history**		
	Diabetes before the age of 55	5 (16)
	Heart attack before the age of 55	6 (19)
	Maternal history of hip fracture	2 (6)
**Highest level of education**		
	High school	6 (19)
	Trade school	2 (6)
	College	7 (22)
	University	7(22)
	Graduate school (MSc, PhD)	7 (22)
	Professional degree (MD, MBA)	1 (3)
**Annual household income**		
	<CAD $20,000	2 (6)
	CAD $20,000-CAD$49,999	6 (19)
	CAD $50,000-CAD $79,999	12 (38)
	>CAD $80,000	10 (31)
	Prefer not to say	2 (6)
**Skill with computers**		
	Nonuser	1 (3)
	Novice	6 (19)
	Intermediate	19 (59)
	Expert	5 (16)
**Use of a computer**		
	Daily	28 (88)
	Weekly	2 (6)
	Monthly	0 (0)
	Rarely	0 (0)
	Never	1 (3)
**Use a mobile phone or tablet**		
	Daily	22 (69)
	Weekly	0 (0)
	Monthly	0 (0)
	Rarely	0 (0)
	Never	12 (31)

^a^Percentages may not add up to 100% due to incomplete surveys or multiple answers.

We included 32 adults over the age of 50 living in southwestern Ontario who had been diagnosed with a chronic disease that could be prevented with physical activity. We excluded individuals who could not speak or read English and who had contraindications to physical activity according to the Physical Activity Readiness Questionnaire [60].

### Thematic Analysis: User Perceptions

Focus groups were conducted at the end of each study group. We chose to do a high-level thematic analysis with the purpose of examining and recording the patterns in our data [66]. We chose thematic analysis because it helped us organize the data into themes to offer a rich description of participant experiences. Thematic analysis goes further than just counting phrases or words in a text and works to identify the implicit and explicit ideas of the data [67]. Borrowing from the grounded theory approach, we did not identify any preconceived theories before starting data collection on the usability or usefulness of the wearable activity trackers for older adults with chronic disease [68]. This gave us the opportunity to incorporate fresh viewpoints from the diverse settings of participant lives, rather than from our own perspectives as heavy users of technology. The final dataset used in the thematic analysis included participants’ written notes, researcher observations, and the recorded and transcribed focus group discussions.

The TAM identifies two key beliefs as the primary reason for behaviors that encourage computer and technology acceptance: *perceived usefulness* and *perceived ease of use*, with the idea being that if people think that using a technology will make their life easier or think a technology is easy to use, then they will be more likely to adopt the technology [69]. In particular, TAM identifies that using technology, both new and established, is associated with behavioral intention, specifically that people form intentions to use technologies when they have a positive feeling about the technology in question. TAM identified the following areas that can influence adoption: external variables, perceived usefulness (U), perceived ease of use (e), attitude toward using (A), behavioral intention to use (BI), and actual system use. It postulates that BI = A + U and stated that people form intentions about using new technologies based on how they perceive the technology will improve their performance.

Our data were coded and analyzed in NVivo (QSR International) in 3 stages. For the initial stage of analysis, 1 independent researcher (KM) coded data by briefly summarizing each line and then paragraph of data. In the second stage of coding, the codes were combined into themes by 2 researchers (KM and KG). In the third and final stage, each theme was populated by representative quotations (KM and KG).

## Results

### User Testing

We recruited a purposive sample of 32 participants aged between 52 and 85 years (mean 64 years; Table 2). Participants were included if they were over the age of 50 and self-reported a diagnosis of one of the following: hypertension, hyperlipidemia, diabetes, osteoarthritis, rheumatoid arthritis, and osteoporosis (including a history of non-traumatic fracture of the wrist, spine or hip). We also included participants who had a significant family history of cardiovascular disease or diabetes (a parent, sibling, or child has developed cardiovascular disease or type E diabetes before the age of 55) and a parental history of hip fracture.

Because our goal was to explore acceptability, which is based on participant’s perceptions of usability and usefulness, a sample size of 32 was adequate. We also continued sampling until data saturation was reached and no new ideas or issues were identified [70].

**Table 3:** Participant experience questionnaire (answer averages) evaluating the use of wearable activity trackers.^a^

No	Feedback	Withings	Fitbit Zip	Jawbone	Pedometer	Shine	Average
1	Overall, I was satisfied with the activity tracker.	3.39	3.57	3.33	1.77	3.37	3.09
2	Using the activity tracker helped me set activity goals.	2.90	3.13	2.93	2.55	3.13	2.93
3	Using the activity tracker helped me reach my activity goals more rapidly.	2.81	3.03	2.67	2.19	2.80	2.70
4	Using the activity tracker helped me to be more active.	3.00	3.20	3.10	2.65	3.13	3.02
5	Using the activity tracker made it easier to be more active.	2.84	3.00	2.87	2.35	2.83	2.78
6	Using the activity tracker supported me in managing my disease.	2.61	3.00	2.60	2.23	2.70	2.63
7	I found it easy to learn to operate the activity tracker.	2.84	3.27	3.13	2.84	3.00	3.02
8	I found the activity tracker to be clear and understandable to use.	3.06	3.40	3.07	2.90	2.90	3.07
9	I found the activity tracker to be flexible to work with.	3.00	3.37	3.23	2.42	3.03	3.01
10	Overall, the activity tracker was easy to use.	3.03	3.43	3.43	2.74	2.97	3.12
11	People who influence my behavior would think I should use the activity tracker.	2.65	2.93	2.87	2.68	2.86	2.80
12	People who are important to me would think I should use the activity tracker.	2.74	3.00	2.97	2.68	2.97	2.87
13	I have the technology necessary to use the activity tracker	3.45	3.90	3.43	3.65	3.50	3.59
14	I have the knowledge necessary to use the activity tracker.	3.26	3.67	3.47	3.90	3.33	3.53
15	The activity tracker was compatible with other systems I use.	2.84	3.53	3.23	2.47	3.23	3.06
16	I am very knowledgeable about my physical activity needs.	4.00	4.17	4.03	3.90	4.03	4.03
17	I understand how to use physical activity to manage my health problems.	4.06	4.17	4.03	4.13	4.07	4.09
18	The activity tracker was comfortable to wear.	3.87	4.13	3.70	3.13	4.10	3.79
19	The activity tracker accurately tracked my physical activity.	3.48	3.50	3.67	1.77	3.77	3.24
20	Average by tracker (SD)	3.15 (0.43)	3.44 (0.40)	3.25 (0.40)	2.79 (0.67)	3.25 (0.44)	

^a^Scoring was as follows: 1=strongly disagree, 2=disagree, 3=neutral, 4=agree, and 5=strongly agree.

Of the 32 participants, 30 completed the testing of all devices; 2 users dropped out after testing the pedometer (initial stage), citing acute viral illness. All the wearable activity trackers tested had moderate acceptability, with the standard pedometers having the lowest acceptability to users (Table 4).

In general, all the wearable activity trackers tested had a similar score for each item of the TAM questionnaire and were rated higher than the standard pedometer in all items but 13 and 14, both of which are related to baseline knowledge of the technology. This coincides with the exit interview ratings, where participants constantly rated the standard pedometer as their least preferred option.

The language participants used about wearable activity trackers was also notable. Participants would pick a favorite device, and stick with that device, even if through later discussion they identified more negatives than positives about that device. The common reasons for liking or disliking a device are how they looked, be it for subtle or fashion-based factors, and ease of use. The participants’ perceived comfort level with the devices was another notable aspect of this study that drove how participants ranked the devices. We also found that the wearable trackers performed highest on items assessing ease of use, namely, Item 10 “Overall, the activity trackers were easy to use” and Item 14 “I have the knowledge necessary to use the activity tracker.” The highest rated item was Question 16, “The activity tracker was comfortable to wear.” This was reflected by low scores for Item 7 “I found it easy to learn to operate the activity tracker” for the Withings and pedometer (2.84) compared with the Fitbit (3.27), Jawbone (3.13), and Shine (3.00), which contributed to decisions to purchase or not purchase (Table 3).

After testing the devices, none of our participants planned to purchase a Mio or Sportline pedometer, whereas 22 of the 30 participants who completed the study said they would purchase a wearable activity tracker. The participants were asked which device or devices they would potentially purchase, and after trying the devices, 30% (N=32) felt they would buy a Jawbone Up 24, 30% would purchase a Misfit Shine, 33% felt they would buy a Withings Pulse, and 40% felt they would buy a Fitbit Zip. At the completion of the study, 73% (22/30) felt they would purchase a tracker and 67% (20/30) felt they would purchase a device for a friend or family member. Those who felt they would not purchase a tracker included reasons of cost, lack of interest, and complexity of the devices. Tables 5 and 6 present the positive and negative associated with individual fitness trackers, respectively.

**Table 4:** Mean participant acceptance scores from the participant acceptance questionnaire for each wearable activity tracker assessed.^a^

App	Mean score (SD)
Pedometer	55.7 (10.2)
Fitbit Zip	67.6 (15.8)
Jawbone Up 24	65.8 (19.1)
Misfit Shine	64.7 (13.7)
Withings Pulse	62.9 (13.8)

^a^Minimum score was 19 points; maximum score was 95 points (N=30).

**Table 5:** Positive statement associated with individual fitness trackers.

App	Positive statement
Pedometer	“I just want a step counter, I don’t care about the rest of the stuff”
Fitbit Zip	“The Fitbit I still say is the easiest. I actually got an email that tells you your weekly progress, and I really like that.”
Jawbone Up 24	“I liked the sleep data a lot, and I found the Jawbone easy to use. I found it really easy, it was light.”
Misfit Shine	“I really liked that I could wear it in the water
Withings Pulse	“A lot of people say they don’t like the Withings, but I really did, I thought it was great”

**Table 6:** Negative statement associated with individual fitness trackers.

App	Negative statement
Pedometer	“The very first one, the pedometer was unbelievably off. I would take one step, and it would count 10.”
Fitbit Zip	“It was too small, I was scared I would lose it”
Jawbone Up 24	“Jawbone, see look, this is by far the easiest to put on and off but once it's on it can get a little annoying”
Misfit Shine	“The Shine I thought was just the most unintuitive, poorly constructed system”
Withings Pulse	“The reason I didn't choose it is because I was afraid that over time it would all break.”

### User Perceptions: Thematic Analysis

In this study, 4 overarching themes emerged to describe how acceptable wearable activity trackers are for older adults with chronic disease. The first theme is that new and emerging consumer health technologies are likely to be outside the older user’s perceived comfort zone. However, the second theme is that after a brief trial period, users can appreciate that wearable activity trackers improve self-awareness and goal setting. The third theme was that wearable activity trackers are ultimately more useful as motivators than as quantifiers. The final and fourth theme was that older adults are unlikely to adopt wearable activity trackers if the trackers are not sold and managed as health care devices.

#### Theme 1: Adoption Within a Comfort Zone

There is a perception that the navigation of devices and apps requires technological know-how that is often absent in the older adult and elderly population. Modifications of the TAM have identified technical know-how as a form of self-efficacy that influences a user’s *perceived ease of use* and *attitude toward using* [69,71,72]. It was clear that improvements could be made to the devices and associated apps to make them more accessible for older adults. This was reflected by many of our participants in the comments they made about the devices—often relating that they were “not built with us in mind,” that they were created “for someone younger,” and that devices needed a more “tech-savvy” user. An issue brought up by each group was that there were no instruction manuals, which prevent them from feeling comfortable with the various devices.

I wonder if people looking at getting one would see younger people wearing these, but that might be a deterrent, saying “well, that's only for young people.”

Female, 58 Group 2

I think the right person is someone who has patience and a real understanding that this person doesn’t get it because they’re simple or stupid or whatever, it’s only because it is like a foreign language to them. So for our generation, they need to back it up and simplify the steps.

Female, 60, Group 2

TAM additionally identifies the lack of instructions as a barrier to a person adopting, because it is a barrier to *actual system use* and can imply to the user that they should be able to use the device and that difficulties are personal failing. In addition to learning to use the device, users must also learn to speak the language of the device, including terms such as “active time,” “1000 points,” and “link with Bluetooth.”

If you look at that little Fitbit, it doesn’t have any instructions with it. That almost did me in. I waited for a child to come along. But I also had to take into account the feelings, like you don’t feel, you feel really limited, you know you feel really badly when you can’t figure it out.

Female, 60 Group 2

I couldn’t figure out how to get steps, it kept giving me some percentage of my daily activity and I couldn’t figure out how to get the steps. So I was told to go online. Of course that seems so intuitive, but for me it wasn’t. The fact that they don’t come with written instructions I think is a real downside.

Female, 58, Group 1

A more practical consideration is comfort, identified by the TAM as *perceived ease of use,* specifically in this study how the device looks, with some participants preferring wristbands and others preferring to clip devices to a belt or bra. This also goes to *actual system use—*if the participants felt there was a potential to lose or break the device, they were less likely to be comfortable with adopting one.

I don’t know which I found most comfortable. I might take the Jawbone because it would stay on. As opposed to the Fitbit I’m not sure if the Fitbit, the way it’s designed there would stay on, and I had trouble clipping on some of the other ones—I didn’t trust they wouldn’t fall off.

Female, 58, Group 2

...[The Jawbone Up 24] was a really beautiful object so you have to kind of get behind that specific look. I like Italian rubber design jewelry so I didn’t mind that this is a nuisance to take off and on cause it’s a cool look. If I’m looking for what I feel comfortable with wearing, I would like a fashion statement.

Female, 67, Group 1

The wearable activity trackers may be less useful for individuals less familiar with mobile technology. For this group, simpler devices with clear displays should suffice.

None of my friends [aged] 80-90 that I play cards with would, they were interested in what I was wearing, but they weren’t interested in it for themselves, they don’t care how many steps they take, they sit most of the time. We read and play cards. We do a bit of exercise, we walk a bit, but as far as wearing one of the simple devices, maybe, but I found those that are more complicated they don’t have the iPad, no computer, but they couldn’t even use it so, we’re out.

Female, 85, Group 1

#### Theme 2: Self-Awareness and Goal Setting: Knowing Where You Are and Where You Want to Be

The greatest advantage to wearable activity trackers is that they help participants become more aware of their activity levels. TAM identifies *behavioral intention to use* as one of the key attitudes to adoption. If a participant wanted to make lifestyle choices, he/she is more likely to identify a *perceived usefulness* of a device, which increased their intention to use it and eventually adopt the technology. In all groups, participants had been asked by a physician to exercise more, and agreed that they needed to be more active.

It was more informative than motivating, because I had my own agenda that my doctor set out for me to do.

Female, 55 Group 3

I’m just interested in the number of steps and exercise really. As far as living healthy, I think we all know what we’re supposed to eat, what our blood pressure should be at and all these sorts of things.

Male, 85, Group 1

However, even if participants thought they were active, they either wondered or worried that they were not as active as they should be.

At least it was telling me something, maybe not what I always wanted to know. I didn’t care what it said. I just knew I had it on. So I wanted to try to be more active. I didn’t care what the numbers said but I have to admit that when I did see the numbers I was like, wow, or a couple of days it was like, whoa.

Female, 56 Group 3

I like the interactive part of these. I don’t really care about the details, and if I’m gaining or losing 500 steps, because I know now I’m not doing enough steps at all, so I had a eureka moment when I thought I need to notch this up and stop being so lazy.

Female, 67, Group 1

Triggers from the activity tracker such as a vibration to alert the user after a period of inactivity (Jawbone Up 24) also increased awareness more than motivation. TAM suggests awareness as a motivator to adoption, falling closely in line with *behavioral intention to use,* as well as with the lead-in to the TAM, *external variables.* If a person is driven to improve his/her health that influences his/her intentions, it leads to potential openness for adoption. However, this growing motivation does not necessarily translate into increased physical activity.

It’s not a motivation, it’s an awareness. I had it set to inactivity buzz every 15 minutes and at times I wouldn’t get up but it was enough to motivate me to realize I just hadn’t moved.

Female, 62 Group 3

Well it reminded me, but it didn’t get me moving. I was working on a computer, and it would buzz, lying down watching a program. I think I had it set for every 20 minutes to vibrate so it lets you know that you’re inactive, but what I did after the reminder to move was up to me.

Male, 62, Group 2

#### Theme 3: By the Numbers: Purposes of Data Tracking

Participants were less interested in being motivated by the activity tracker than in being motivated by the self-awareness gained from data collected by the tracker. The *perceived usefulness* and *behavioral intention to use* help the users to find clarity around real and perceived activity levels. This clarity was a step toward finding an intrinsic motivation to become more active.

I think the issue is always how do you motivate yourself to do things you know are good for you, so that was part of how I was thinking about it, not just for myself. I’ve been in a really heavy workload so I've been sitting a lot so it actually shocked me to know I only do 2000 steps a day, so that was super motivating for me.

Female, 67, Group 1

I was diagnosed years ago with osteoporosis so I’ve always felt like I need to have at least 3 hours of activity in the bone bank, and once I retired I thought not good enough, I need to have 4 or 5 hours in the bone bank. I found using these helped. That, well, my husband said I was developing obsessive compulsive disorder because I was constantly moving, I’d run up and down the stairs, or I’d dance with a grandchild or something and I’d look down. So it was hugely motivating.

Female, 62, Group 3

Self-awareness translated into motivation when it made activity a game or competition for some participants. For participants, the “goal” of 10,000 steps seemed to matter less than being aware of how much, or how little activity they got, compared with where they wanted to be.

I was trying to get to 10,000 steps and I did, a few times, it was fun, and you could see most of the different things...you know my activity level is higher at this time and lower at that time, and let’s run up and down stairs a couple times, and I got another 2 minutes. It was a lot of fun.

Female, 58, Group 3

The goal was 10,000 steps. These trackers really let you know how much more than your daily routine you really need to put in to get to that goal. Doing your normal day-to-day thing, you’re not even close.

Male, 65, Group 2

As demonstrated by our oldest user, for users who are isolated in their home, the wearable activity trackers may help users compete against themselves more easily by automatically collecting data and tracking it over days and weeks.

Very definitely, you know you compete with yourself. I have no one else to compete with. This winter has been hard, for going outside and walking. I used to be able to walk, or I used to be a swimmer, and now I’ve become a couch person...I’ve done the track in the house, I run up and down the stairs, down to the basement and I walk my driveway a couple of times cause it’s long. I try and I compete with myself and I know that I sit or lie down much too long. But when you’re over 80 I think that’s excusable.

Female, 85, Group 1

#### Theme 4: The Future of Wearable Activity Trackers as Health Care Devices

Overall, participants generally enjoyed trying out the wearable activity trackers (Textbox 1). In the exit focus groups, participants were asked what they thought the opportunities were for the future of wearable devices. The responses varied, but one of the statements commonly repeated was the need for the health sector to get involved in promoting physical activity trackers to patients as a possible way to improve their health.

I think the next step should be a handout in consultation with medical groups like pharmacies and in partnership with provincial and federal groups and maybe even activity groups like the YMCA to come up with a really effective comprehensive, simple pamphlet pointing out the importance of what you do in the hours you’re awake. I think then we’ll need some kind of financial incentive because there are limitations to people being able to afford a tablet and the device.

Male, 78, Group 1

In Canada, physical activity trackers are not taxed if bought with a prescription. Several of our participants also stated they wished the devices were available in pharmacies because they did not go into the electronics stores where the devices are traditionally sold.

But if someone can guide you through it, I think any of them, once you start using them you would probably use it. But I wouldn’t go to Best Buy I wouldn’t have thought to go to best buy. If it’s for my health, I would think to go to a pharmacy.

Female, 52, Group 3

There was also a noted desire to learn about the devices from someone in health care. The participants were interested if their doctors or other health care professionals would be interested in the data provided from the devices. There was also a noticed interest in pharmacies carrying the devices, and having pharmacists able to explain how to use them, similar to how health-monitoring systems such as blood glucose meters or blood pressure meters are explained by pharmacists. Several times a barrier to learning was that the participant asked older/adult children for help using the device, and was met with impatience and frustration.

My daughter was no help at all. She just kept saying it’s stupid, I don’t have time.

Female, 59, Group 2

**A brief summary of participants’ experience in trying wearable activity trackers in one word.**

Negative words:

- Annoying
- Challenging
- Stressful
- Hard
- Frustrating

Neutral words:

- Instructive
- Learning experience
- Interesting
- Informative
- Fine
- Educational
- Life
- Experiential

Positive words:

- Fun
- Exciting
- Motivational
- Comfortable
- Motivational

## Discussion

Running from January 2014 to June 2014, our study included 32 older adults living with chronic illness who were trying wearable activity trackers for the first time. We found that the study participants generally enjoyed using the widely available trackers and even preferred them over the standard pedometer. Our participants also found the trackers to be useful in promoting self-awareness and motivation. However, it should be noted that at the time of this study, these trackers were an emerging technology; thus, participants often felt that these devices were too new to be comfortable with. When asked, our participants suggested that wearable activity trackers should be recommended for health care rather than entertainment. To meet this final theme, it was suggested that the devices be available at pharmacies and sold alongside blood pressure and blood glucose meters with the standard health-related tax exemptions or credits.

At the beginning of the study, the initial feedback during recruitment from several older adults and seniors was that they did not use mobile phones and tablets and were unsure whether they were the right choices for the study. Throughout the study, however, we found that these participants were often the ones who had the least trouble adapting to new technology, and many times knew more about new technologies than they thought they did. Frustration often came from the apps during use, and the lack of clear instructions for installation rather than understanding and using the technology.

Research into wearable activity trackers is a new area but it is closely tied to the growing body of research in mHealth. Several previous studies have evaluated the use of mobile phones in supporting health care and public health interventions, particularly in the collection of data for health research [73,74]. The area is rapidly growing, with the number of mobile phone health and fitness apps growing from 7000 in 2010 to over 40,000 in 2013 [75,76]. Studies assessing specific functionality of mobile phones have recently looked at digital diaries in symptom research, short message service texting to manage behavior change, and the use of mobile phone records against traditional paper records in drug trials [77-80]. Free et al [81] identified several key features that give mobile phones advantages over other communication technologies, including portability, continuous data, and sufficient computing power to support multimedia-based interfaces. Other reviews also offer more information about how to incorporate mobile phones and other small devices in health and clinical practice [82-84].

Traditional pedometers digitally monitor and track basic physical activity. They are essential to programs that recommend a specific daily step count. Many of the participants we interviewed cited comfort and experience with using pedometers because they are appealing to older adults uncomfortable with technology. However, long-term tracking requires manual data entry, which hinders engagement of the user. The wireless transmission of data allows for timestamps, measurement of intensity, frequency and duration ideally without significant input from the user, and then sending the data automatically to devices that report back to the user such as mobile phones, tablets, and computers [85]. It was also clear from our studies, that when given the choice, participants preferred a newer wearable activity tracker over a traditional pedometer.

Some of the most important lessons we learned over the course of this study were related to how our participants were using the new technologies in their daily lives. We heard from several participants that adult children were encouraging them to get a mobile phone or had bought or handed down a tablet. In one case, an adult child had recently gifted a participant with a Jawbone Up 24. We found that often our participants were not aware of how much they used the new apps and technologies, citing that they just used their tablet for email and simple card games. As we spent more time with each participant, we saw them browsing for health information, using tablets to check the stock market, checking Facebook, tracking calories, and playing new games.

More research needs to be carried out to fully understand the best practices for designing wearables for older adult populations. There is significant potential for stakeholders to promote and use wearables as a tool to encourage, motivate, and assist older adults in improving their health. Future wearables could benefit from including a simple paper-based instruction manual that clearly addresses set up, how to use the device. and basic problem solving. This would provide knowledge to older adults in a medium they are familiar with, which has potential to increase adoption. There is also significant potential for designing wearable fitness trackers in a way that older adults can benefit from both on the device itself and in the accompanying app. Displays should consider using large, high-contrast text with large light-on-dark letters and numbers to allow for easier viewing. In addition, allowing access to device knowledge on both a computer and a mobile app would allow older adults to access data in a more familiar way, in terms of comfort with technology and by allowing them to view results on a bigger screen. Waterproof design decreases worry about the fragility of the device if it is forgotten, and accidentally damaged by doing dishes or the laundry, and also allows older adults to use it in the water-based activities that are commonly recommended by health care providers as part of a low-impact way to increase physical activity.

The primary limitation of our study is that we gave participants the devices for a purposely short period, with the goal of getting initial impressions. There is significant opportunity to test wearable devices for longer periods to determine long-term adherence as well as to test the devices with a population that is more universally sedentary. We also purposely ran our study in the winter to spring months, when many of our participants self-identified as less active than they would be in the summer. A key driver of this project was to determine if awareness in their actual activity times would increase or decrease dependent on awareness despite there being challenges to simply “going for a walk.” Given the qualitative nature of our inquiry, we were also careful to not focus on the effects of the wearable activity trackers on step counts.

In conclusion, our goal with this study was to determine the acceptability and usability of the various wearable fitness trackers for adults over the age of 50 and whether the devices may be something these adults would be interested in, acknowledging that we are in an early adopter phase with this technology. We worked with 30 adults (all aged >50) with a chronic illness to determine how they perceive the usability and acceptance of wearable fitness trackers. Overall, we found that there was a meaningful potential in using wearable fitness trackers as a multifaceted intervention to help older adults become more active. If health professionals can help older adults become more aware of wearable activity trackers, there is potential for adoption, and through adoption, for creating more awareness of physical activity levels. The benefits of mHealth technologies, specifically in this case wearable activity trackers, lie in their potential to overcome barriers between patients, clinicians. and researchers through giving users independent insight into the realities of their physical fitness, which translates into their awareness of their active times and arguably most significantly, awareness of user’s own real levels of physical activity.

## Abbreviations

MET: metabolic equivalent of task
TAM: Technology Acceptance Model
